# Unveiling CD177: a key player in tumors, autoimmune diseases, and inflammatory disorders

**DOI:** 10.3389/fimmu.2025.1653587

**Published:** 2025-10-09

**Authors:** Dongmei Yang, Xiaobin Hu

**Affiliations:** Clinical Laboratory, The Second People 's Hospital of Yibin, Yibin, Sichuan, China

**Keywords:** CD177, clinical value, neutrophils, tumor, inflammatory, immunity

## Abstract

CD177, also referred to as human neutrophil antigen NB1 or polycythemia rubra vera-1, is a glycosylphosphatidylinositol-anchored glycoprotein with a molecular weight of approximately 58−64 kDa, predominantly expressed in neutrophils, neutrophilic myelocytes, and metamyelocytes. While extensive research has focused on the role of CD177 in neutrophils, where it is implicated in transendothelial migration, cellular viability, and bactericidal activities, its functions outside of neutrophils remain largely unexplored. Under inflammatory conditions, CD177 expression on neutrophils is significantly upregulated, facilitating their migration to sites of inflammation. CD177^+^ neutrophils have been shown to accumulate in inflamed tissues and modulate the release of inflammatory mediators and the formation of neutrophil extracellular traps (NETs), correlating with the severity of inflammation in conditions such as inflammatory bowel disease (IBD) and acute respiratory distress syndrome (ARDS). An imbalance in CD177^+^ neutrophils has been identified as a critical pathogenic mechanism in vascular inflammation, tissue necrosis, and systemic lupus erythematosus (SLE). Furthermore, CD177 expression on neutrophils, epithelial cells, and regulatory T cells in solid tumors has been associated with tumor invasion, disease stage, therapeutic responses, and patient survival in various cancers, including gastric, breast, and colorectal cancer (CRC). Nevertheless, elucidating the intricate mechanisms underlying CD177’s role in these diseases remains a significant challenge. In light of these findings, we present a comprehensive review of recent literature concerning the role of CD177 in tumors, inflammatory processes, and autoimmune diseases, with a particular focus on its mediating effects on neutrophil recruitment, transepithelial migration, and the activation of CD177-positive neutrophils, along with the functional implications of CD177 expression beyond neutrophils. A deeper understanding of CD177 may pave the way for the development of novel therapeutic strategies and the assessment of disease prognosis.

## 1 Introduction

*The leukocyte differentiation antigen CD177*, initially identified as NB1 (1, 2)and subsequently renamed as CD177, is a glycoprotein of ∼60 kDa attached to the neutrophil surface via a GPI linker (3, 4). CD177 is present on 40–60% of neutrophils in most donors, with a range extending from of 0 -100% (5). The reason for the expression of CD177 in some neutrophils but not others is incompletely understood. Due to the absence of a transmembrane domain, CD177 is incapable of independently transmitting intracellular signals. The biological functions of CD177 encompass the modulation of human neutrophil migration, the formation of neutrophil extracellular traps (NETs), and the presentation of antigens in various immune disorders.

Additionally, CD177 serves as a valuable flow cytometry (FCM) marker for myelodysplastic syndrome (MDS) (6–8). Currently, extensive research has been conducted on CD177 in the context of inflammation and immune diseases, including SLE, Kawasaki disease, and COVID-19 (9–11). Furthermore, CD177 exhibits distinct roles in influencing the clinical significance and prognostic value across multiple types of cancer.CD177 has been shown to significantly correlate with a favorable prognosis in breast cancer and gastric cancer, while exhibiting a negative correlation with prognosis in ovarian cancer (12–14). Notably, the functional role of CD177 expressed on tumor-infiltrating regulatory T cells and epithelial cells within tumors has also been found in breast cancer and renal clear cell carcinoma (15). However, the precise expression patterns and mechanisms of action of CD177 across various organs and tissues remain inadequately understood. Therefore, further investigation into the functional gaps and regulatory mechanisms of CD177, particularly its involvement in tumor immunity and inflammatory processes, is imperative. This review primarily aims to elucidate the biological characteristics and functions of CD177, while also exploring its potential as a biomarker for disease prediction and diagnosis, and analyzing the underlying molecular mechanisms. These insights not only enhance our understanding of CD177’s functional role but also underscore its potential application in clinical personalized treatment strategies in the future.

## 2 Overview of CD177

The *CD177* gene is a member of the leukocyte antigen 6 (Ly-6) superfamily (16), with a molecular weight ranging from approximately 58 to 64 kDa, and is located on chromosome 19q13.31 (17, 18). This gene exhibits polymorphism, with *HNA-2a* and *NB1* representing its alleles. The glycoprotein CD177, which is also referred to as HNA-2a or NB1, is a membrane-bound receptor protein encoded by the *CD177* gene. It consists of 437 amino acids and is classified as a leukocyte differentiation antigen (19, 20). CD177 is predominantly expressed on granulocytes, with lower expression levels observed in monocytes and lymphocytes. It is primarily localized in the bone marrow and peripheral blood, playing a crucial role in the onset and progression of various diseases.

## 3 Main functions of CD177

Under physiological conditions, CD177 shows heterogeneous expression. As a high-frequency antigen, CD177 is expressed on subpopulations of neutrophils in the peripheral blood of approximately 97% of healthy individuals and presents on 40–60% of neutrophils in most donors, with a range extending from 0 to 100% (5). The percentage of CD177^+^ neutrophils varies among individuals. In 3-5% of the general population, CD177 is completely absent from all neutrophils, with these individuals commonly referred to as CD177-null (21). In addition, CD177 was also found to be expressed in different tissues and organs under various conditions including epithelial cells and regulatory T cells (15). Genesets uncovered that high CD177 expression was associated with keratin filaments, immunoglobulin complexes, and the regulation of lymphocyte activation (22), but the mechanism is rarely elucidated in the literature. Therefore, we focus here on the function of CD177 in neutrophils. Flow cytometry (FCM) is typically used to measure the expression of CD177, including the percentage of CD177^+^ cells and the mean fluorescence intensity of CD177 (CD177^+^ MFI), as well as to distinguish between CD177^+^ and CD177^-^ neutrophil subsets. CD177 deficiency constitutes a prevalent human phenotype, attributed to genetic variations such as c.1291G>A, which leads to protein instability or the lack of membrane expression while preserving the ability to bind to PR3 or immunoglobulin G (IgG) (23, 24). It interacts with the human IgG Fc receptor, and genetic variations within this context can significantly influence antibody-dependent cell-mediated cytotoxicity (ADCC) function, thereby presenting the potential to serve as a biomarker for specific diseases (25).

Under pathological conditions, high expression of CD177 facilitates neutrophil activation, adhesion, migration and the release of inflammatory cytokines by interacting with β-2 integrin, platelet endothelial cell adhesion molecule-1 (PECAM-1), protease3. CD177 also regulates the formation of NET to mediate immune and inflammatory responses. Integrins function as cellular adhesion receptors and are instrumental as “key messengers” in the bidirectional signaling between intracellular and intercellular compartments (26). CD177 interacts with β-2 integrin heterodimers, specifically ITGAM/CD11b and ITGB2/CD18, mediating the external activation of neutrophils induced by tumor necrosis factor-alpha (TNF-α), which encompasses degranulation and superoxide generation (6, 27). At the same time, the activation of β2 integrins also promotes neutrophil adhesion and inhibits migration. Internally, CD177 inhibits the internalization of β-2 integrin and attenuates chemokine signaling, thereby promoting neutrophil adhesion at the expense of migration. CD177 binds to PECAM-1 (also known as CD31), an adhesion molecule primarily located at the junctions between endothelial cells, is essential for neutrophil transendothelial migration *in vitro*, a process that is dependent on the presence of Ca²^+^ (28).CD177 promotes transendothelial migration of CD177^+^ neutrophils and recruits them to exert immune effects at sites of inflammation by binding to PECAM-1. However, its significance in the migration and recruitment of neutrophils during bacterial infections *in vivo* appears to be less pronounced (29). There is still a lack of direct experiments exploring the interaction between CD177 and β2 integrins, with only indirect evidence describing the impact of their interaction on neutrophil adhesion, migration, and recruitment.

Furthermore, CD177 is capable of binding to PR3, an autoantigen found on the membrane surface of distinct neutrophil subsets (30, 31). The interaction facilitates neutrophils to enhance the integrity of endothelial cell junctions during vascular extravasation, which plays a vital role in maintaining vascular integrity (32). CD177 forms a complex with membrane-bound mPR3, necessitating the involvement of GPR97. GPR97 interacts with mPR3 through two different domains of its extracellular to complete the assembly of macromolecular complexes composed of CD177, GPR97, PAR2 and CD16 B. The complex promotes neutrophil activation and produces superoxide by stimulating the G protein-coupled receptor PAR2 (33). The formation of the CD177/mPR3/GPR97 complex represents a crucial step in the activation of neutrophils; its dysregulation may contribute to the onset of inflammatory conditions or autoimmune diseases.

Neutrophils are the most abundant immune cells in circulating blood and function as the primary anti-inflammatory “vanguards” of the innate immune system. Their activities are crucial for maintaining internal homeostasis and influencing the progression and outcomes of various diseases. NET is a net-like structure composed of DNA, histones, myeloperoxidase(MPO), and various granule proteins that are released during neutrophil disintegration. This process, known as NETosis, is a vital mechanism through which neutrophils exert their effector functions. Research (34) has identified CD177^+^ neutrophils as a key functional subpopulation that significantly contributes to bile duct epithelial cell injury via NET formation. Specifically, CD177^+^ neutrophils induce apoptosis in bile duct epithelial cells by releasing NET, a phenomenon potentially linked to CD177’s role in upregulating mitochondrial oxidative phosphorylation and the expression of oxidative stress-related genes. Additionally, NET inhibitors have been shown to effectively reduce CD177 levels on neutrophils and diminish the production of reactive oxygen species (ROS) (35). A report related to extrahepatic biliary atresia (BA) (36), CD177^+^ neutrophils have been observed to exacerbate bile duct injury through NET release, treatment with NET inhibitors has demonstrated a reduction in the number of CD177^+^ neutrophils and ROS production, underscoring the potential therapeutic benefits of these inhibitors. Furthermore, CD177^+^ neutrophils play a protective role in IBD by facilitating IL-22 production and NET formation (37). Higher CD177+ neutrophils are not only associated with the excessive production of NETs, which include ROS, MPO, and peptidylarginine deiminase 4 (PAD4), but also can activate the AIM2 or NLRP3 inflammasome, leading to the release of inflammatory factors such as IL-1β, thereby exacerbating inflammation (38, 39). A protective effect of exogenous CD177 protein(rhCD177/anti-CD177) has been shown to effectively inhibit NETosis and reduce neutrophil-mediated cytotoxicity (39, 40). This inhibitory effect can be contingent upon the heterophilic binding of CD177 to CD31, which induces CD31 and CD177-dependent phosphorylation of SHP-1 (28, 40). Notably, the role of exogenous CD177-mediated NETs in these two studies contrasts sharply with that of CD177^+^ neutrophils in spontaneous NET release. This discrepancy may arise from the binding of exogenous CD177 to specific molecules that potentially affect other mechanisms involved in NETosis on the neutrophil membrane. The inhibitory effect of exogenous CD177 on NET formation appears to be substantially greater than the level of NETs spontaneously released by CD177^+^ neutrophils, thereby highlighting a pronounced inhibition of netosis.

**Figure 1:**
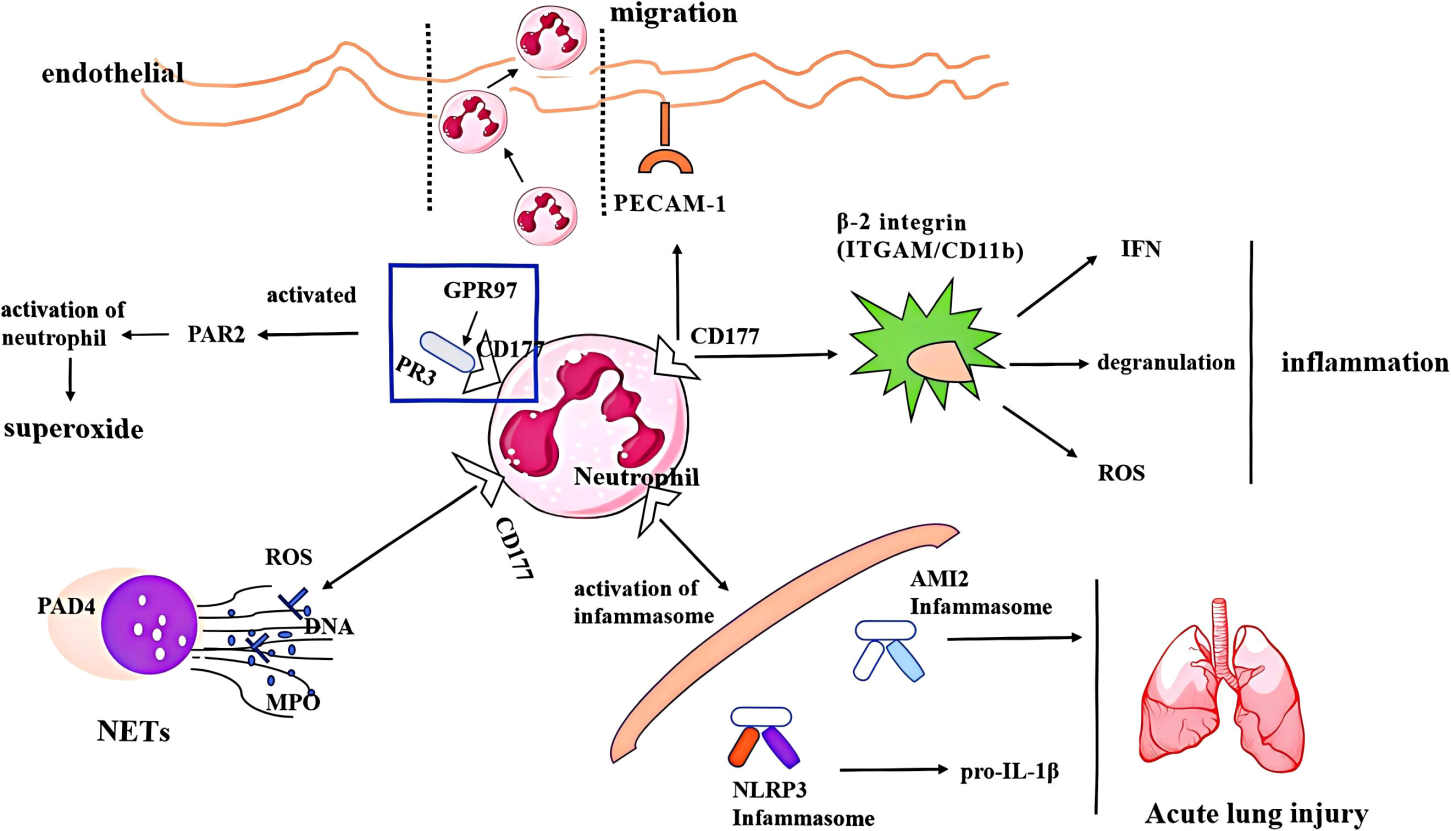
The regulatory mechanism of CD177 on neutrophils, including (1) binding molecules, PR3 and PECAM-1 facilitates CD177+ neutrophil migration, degranulation and release of superpxide;(2) CD177 + neutrophils release ROS and NETs; (2) activating inflammasome and releasing inflammatory cytokines to accelerate lung damage. PECAM-1, platelet endothelial cell adhesion molecule-1; PR3, protease 3; PAD4, peptidyl arginine deiminase 4; ROS, reactive oxygen species; MPO, myeloperoxidase; NET, neutrophil extracellular trap; AMI2, Absent in Melanoma 2; INF, interferon.

Overall, as a surface molecule, CD177 regulates neutrophil-mediated inflammatory responses and exhibits complex immunoregulatory functions through its expression in various tissues and organs, including binding PECAM-1 and mPR3 to facilitate neutrophil migration, degranulation, and release of superpxide, regulating the release of ROS and formation of NETs, as well as activating the inflammasome and releasing inflammatory cytokines to accelerate tissue damage. The primary functional mechanism of CD177 in neutrophils was shown (**Figure 1**). Moreover, the genetic variation of CD177 or the partial expression of CD177 outside neutrophils also shows a strong biological function, which is mainly reported in many tumors. However, the specific expression and mechanism of CD177 in different organs and tissues are not fully understood. Although a large number of studies have reported the relationship between CD177 and NET formation in various diseases, its precise regulatory mechanism is still unclear. The research on CD177 and neutrophil ferroptosis or copper death is still blank. At present, there is still a lack of multi-center and large-sample clinical research on CD177 as a potential biomarker and therapeutic target. These research gaps are the key breakthroughs to further explore the function of CD177 in the future.

## 4 Application of CD177 in specific diseases

As an important functional cell subset to maintain body homeostasis, the CD177^+^ neutrophil population is also closely related to systemic multisystem diseases, including digestive system diseases, respiratory system diseases, reproductive system, immune system, etc.CD177 is directly or indirectly involved in the occurrence and progression of disease by regulating the function of neutrophils, or a small number of CD177 expressed outside neutrophils are directly involved in disease. This review aims to summarize the biological characteristics and functions of CD177, as well as its potential utility as a biomarker across various diseases, including tumors, inflammatory disorders, and autoimmune conditions, as well as to analyze the molecular mechanisms involved and some viewpoints.

### 4.1 CD177 and tumor

CD177 are considered to have an important role in affecting the clinical and prognostic value of various cancers. However, the expression levels of CD177 detected in different sites from multiple tumors are different. Limited literature indicates a correlation between the loss of CD177 expression and poor prognosis in intestinal tumors, such as CRC (22, 41), and gastric cancer (14, 42). Studies (43–45) have found that a high expression of CD177 on neutrophils in tumor tissues from CRC, gastric cancer patients, and a positive correlation with overall survival(OS) and disease-free survival. Furthermore, CD177^+^ neutrophils may serve as an independent prognostic factor for survival outcomes and a potential therapeutic target in intestinal tumors (46). Long et al. (47) and Yu L et al. (48) also demonstrated overexpression of CD177 significantly inhibited the proliferation, migration and invasion of colon cancer cells, which was linked to overall survival. PI3K / AKT and MAPK / ERK signaling pathways are scientific research hotspots that regulate various biological functions such as cell growth, proliferation, differentiation and migration. Which have been widely reported to play an important role in the occurrence, progression and outcome of tumors. We propose that CD177 may serve as an upstream regulatory target for the PI3K/AKT and ERK signaling pathways. High levels of CD177^+^ neutrophils are activated and significantly enriched at the tumor sites where these neutrophils are interacting with tissue, activating particular signaling molecules, leading to the negative regulation of the PI3K/AKT and MAPK/ERK pathways, which may inhibit tumor proliferation, metastasis, and invasion. This presents a promising avenue for future in-depth exploration. In general, CD177 ^+^ neutrophils are new indicators for monitoring the good prognosis of intestinal tumors. In contrast, the proportion of CD177 ^+^ neutrophils in MDS patients decreased, which may be attributed to specific gene mutations, including NPM1 and Runx1 (7) (49).

CD177 is also expressed by solid tumor TI Treg cells, which exhibited distinct transcriptional profiles characterized by enhanced immunosuppressive capabilities and were correlated with poor patient prognosis,including CRC (50), esophageal squamous cell carcinoma (ESCC) (51), hepatocellular carcinoma (HCC) (52), breast cancer, lung cancer, and melanoma (15, 53).CD177^+^ TiTregs may enable positive Treg cell subsets to interact with their ligand PECAM-1, and give these cells different functions. About 22.4% CD177^+^ Treg cells among TI Treg cells in breast cancer and 16.8% in ccRCC (53). These CD177^+^Tregs may represent a promising target for immunotherapy in CRC, particularly in augmenting the efficacy of CAR-T cell therapy. The infiltration of CD177^+^ Treg cells was associated with poor overall survival and reduced response to PD-1 immunotherapy in ESCC. The expression level is higher than that of granulocytes, which express CD177 mRNA (51). Kim et al (15) observed that CD177^+^ Treg cells are fully differentiated and functional Treg cells, which is supported by the fact that CD177^+^ Treg cells are more suppressive than CD177^−^ Treg cells in suppression assays as well as in breast models. Treatment with an anti-CD177-specific monoclonal antibody blocked the suppressive activation of CD177^+^ TI Treg cells. The same anti-CD177 antibody is known to block CD177-mediated neutrophil transmigration (39). In addition, Liang et al. (52)reported that apoptosis-related transcription factor REL drives the differentiation of CD177^+^ Ti Tregs, accompanied by apoptosis and enhanced immunosuppression. Inhibition of CD177 in Tregs can significantly weaken its immunosuppressive function and prevent the progression of HCC.

**Figure 2:**
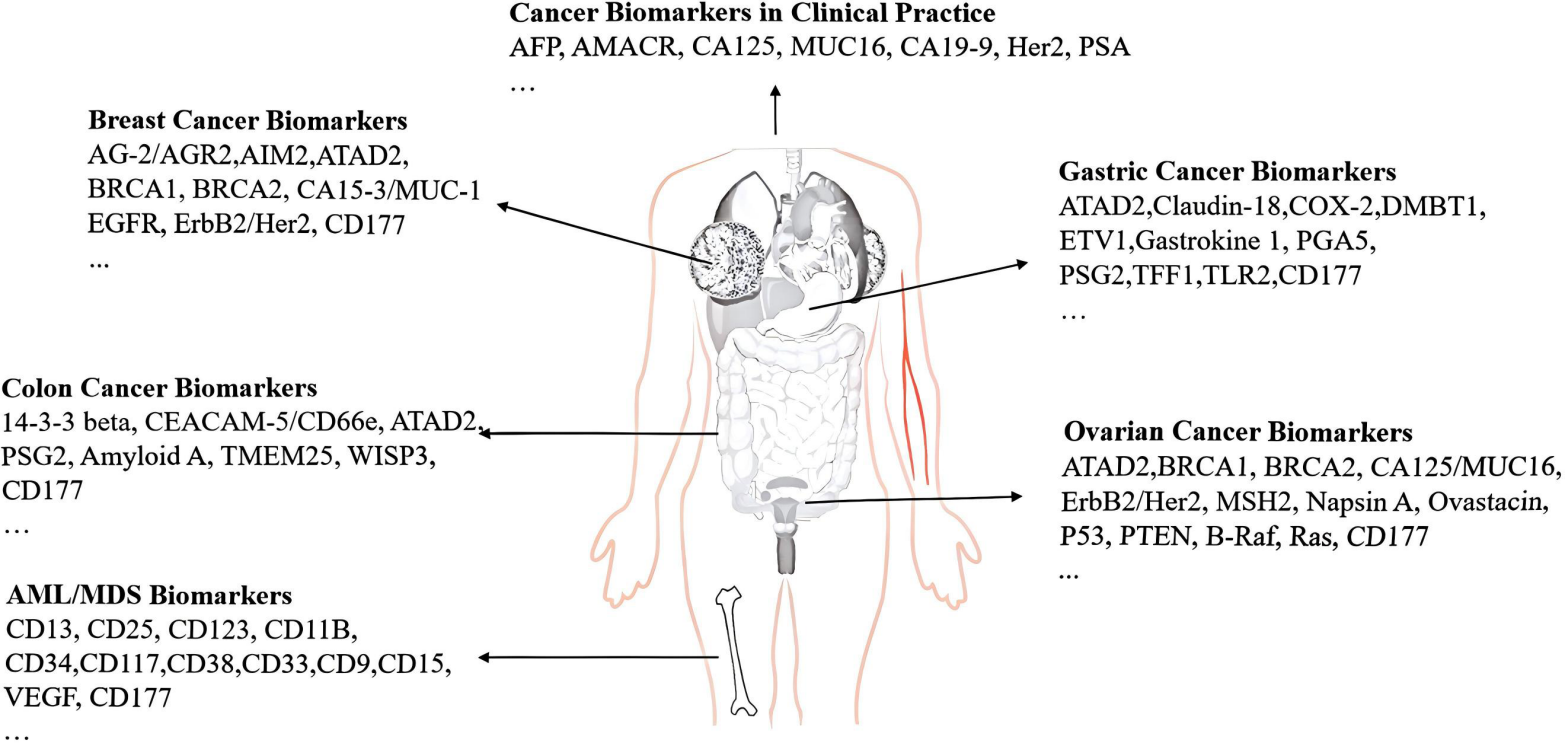
Biomarkers of several common tumors. (1)CD177 is abnormally expressed and is associated with tumor diagnosis and prognosis, including but not limited to breast cancer, gastrointestinal tumors, gastric cancer, ovarian cancer, and hematological malignancies. (2) Additionally, we provide a list of other widely reported biomarkers associated with common tumors for reference.

CD177 was found to be expressed on epithelial cells from the colon, breast, and prostate, as well as immune cell infiltrates from various human tissues, including colon, breast, prostate, liver, lung, lymph node, and spleen from the Human Protein Atlas (54) and Kim et al. reported (15). Low level of CD177 expressed in epithelial cells of breast cancer (12) and cervical cancer tissue (55) predicts the poor prognosis. The mechanism involves regulating the Wnt/β-catenin signaling pathway to inhibit tumor cell proliferation, invasion and migration. But Jiang et al. (13) reported that the proportion of CD177^+^ in ovarian cancer epithelial tissue was increased significantly and exhibited a positive correlation with tumor differentiation, tumor diameter, tumor stage, and sensitivity to platinum-based chemotherapy. Overexpression of CD177 has a negative correlation with poor prognosis, due to the small sample size of the study (only 31 cases), and no long-term follow-up study was conducted; therefore, the conclusion still needs a large multi-center sample. The results from Wang et al (56) are consistent with the view of Jiang et al (13) that OS was inversely associated with increased expression of CD177 mRNA CD177 in pancreatic ductal adenocarcinoma(PDAC) tissues from the Gene Expression Omnibus (GEO) database. Although they failed to quantify neutrophils in tumor tissues, and there is no direct evidence that CD177 expression is only derived from infiltrating neutrophils in tumor tissues, it may also be expressed on other immune cells (such as Treg cells). The value of CD177 for the diagnosis, treatment, and prognosis of PDAC is clear. In general, the expression of CD177 play a dual role for evaluating the prognosis of various tumors, which may depend on the different functions of CD177-mediated neutrophils, Treg cells, and tumor tissue cells. The specific mechanism has not been fully elucidated. If we denote the expression of CD177 on neutrophils as A, its expression on Treg cells as B, and the expression within solid cancer tissues as C, this framework effectively explains the differential expression of CD177 reported in some literature. This dual approach will broaden our understanding of potential therapeutic strategies. We have also summarized several molecular markers associated with common tumors in**Figure 2**.

### 4.2 CD177 and inflammation

CD177 is mainly involved in the inflammatory pathological process by regulating the function of neutrophils. Some studies indicated that CD177 serves as a specific neutrophil activation marker to predict the severity and mortality of COVID-19 (11) (57, 58). The percentage of CD177^+^ neutrophils was increased in the peripheral circulation of patients with new coronal pneumonia, particularly in cases of severe pneumonia (11). CD177^+^ neutrophil demonstrates high accuracy in identifying patients with severe forms of the disease. The results are consistent with another research conducted on the French population, which was also reported that CD177 expression levels were significantly associated with the rate of ICU admissions. Moreover, Potts M et al. (11)observed that CD4^+^ T lymphocyte subsets in COVID-19 patients also expressed CD177, with the expression level of CD177 in CD4^+^ T cells showing a positive correlation with disease severity, underscoring the clinical value of CD177 expression beyond neutrophils. However, little research has been done to explain its specific mechanism.

Studies have found that CD177^+^ neutrophils are mainly enriched in patients with Kawasaki disease (KD) and multisystem inflammatory syndrome in children (MIS-C) (33) (9), especially in the acute stage. Excessive activation of CD177^+^ neutrophils and their pathogenicity may be an important factor in the pathogenesis of KD or MIS-C, and are associated with concurrent cardiovascular risk, but the specific mechanism has not been clarified. In research reports related to acute lung injury (ALI) (38), CD177^+^ neutrophils have been identified as the predominant subpopulation of neutrophils within septic lung tissue. These cells exhibit unique pro-inflammatory and oxidative stress characteristics by regulating the production of excessive NET and the formation of the AIM2 inflammasome, which exacerbates ALI. A similar study also confirmed that a higher percentage of CD177^+^ neutrophils was a positive correlation with serverity among ARDS patients (39). Anti-CD177 effectively reduces neutrophil infiltration and the release of specific inflammatory cytokines, such as IL-1β, while also diminishing the expression of NLRP3, caspase-1, PAD4, MPO, and ROS, indicating that CD177-mediated NET formation and NLRP3 inflammasome formation are involved in ALI/ARDS, which is consistent with the research of Zhang et al (35). In particular, CD177 is implicated in neutrophil activation and the regulation of inflammatory responses in conditions such as sepsis (48, 59)and allergic asthma (60). Neutrophils from septic patients had a higher expression of CD177 (measured as MFI and the percentage of CD177^+^ neutrophils), but a reduction of NET formation was reported by Mulet M et al (59). NET production was negatively correlated with the percentage of CD177^+^ neutrophils. This conclusion is different from the views of Zhang R et al. (34) and Zhou G et al. (37). CD177 plays a dual role in mediating the release of NETs by neutrophils under different inflammatory conditions. The reasons are as follows:(1) NET formation is affected by pH changes. (2)Although CD177^+^ neutrophils are increased in patients with sepsis, the ability of NET release is impaired. (3)CD177^+^ neutrophils release NET dependent on platelet binding; the study found that CD177^+^ neutrophils from septic patients bind fewer platelets (11). Therefore, under different disease states, the outcome of CD177^+^ neutrophils and NET release is not exactly the same due to multiple factors.

### 4.3 CD177 and autoimmune diseases

The imbalance in neutrophil activation prompted by mPR3 binding to CD177 is recognized as a prevalent pathogenic mechanism underlying vascular inflammation and necrosis (61, 62, 63). Based on the combination of CD177 and mPR3, neutrophils can be categorized into two distinct groups (64): CD177-positive/mPR3-high and CD177-negative/mPR3-low. In the context of ANCA-associated vasculitis, PR3-ANCA stimulation leads to CD177-positive/mPR3-high neutrophils producing significantly greater levels of superoxide compared to their CD177-negative/mPR3-low counterparts. Notably, the application of the anti-CD177 Fab clone 40 effectively reduced superoxide production in CD177-positive cells to levels comparable to those observed in CD177-negative cells (30).

In addition to its interaction with mPR3, CD177 serves as a high-affinity heterophilic binding partner for the endothelial cell adhesion molecule PECAM-1. The cross-linking interactions among these three entities have been further elucidated in an additional study (65). This research demonstrated that the interaction between CD177 and PECAM-1 can downregulate the expression of mPR3 on neutrophils, thereby mitigating the activation of neutrophils induced by PR3-ANCA-positive IgG and subsequently reducing glomerular endothelial cell injury. This mechanism is particularly pertinent in the context of Wegener’s granulomatosis (WG) (30), a life-threatening autoimmune disorder. On one hand, CD177 directly interacts with PECAM-1 to facilitate neutrophil activation, thereby enhancing their transendothelial migration capabilities. On the other hand, CD177 also engages with PECAM-1 to mitigate abnormal neutrophil activation induced by PR3-ANCA -positive IgG, as well as to protect glomerular endothelial cells from damage. Although both CD177 and PECAM-1 are involved in trans-endothelial migration, the mechanisms are different. PECAM-1 mediates the migration of neutrophils and tumor cells, which can be independent of CD177, while CD177 enhances the migration ability of neutrophils and Treg cells and plays a synergistic role. In summary, the interaction between CD177 and PECAM-1, along with mPR3, orchestrates the regulation of immune cell activation and migration through the formation of multiprotein complexes and the activation of distinct signaling pathways.

CD177^+^ neutrophils play a protective role in IBD through enhanced IL-22 production and bactericidal activity (66, 67). Zhou et al. (37) has demonstrated a significant increase in the population of CD177^+^ neutrophils in the peripheral blood and inflamed mucosal tissues of patients with active IBD, with increasing production of ROS, NETs, and the release of antimicrobial peptides, producing lower levels of pro-inflammatory cytokines such as interferon-γand interleukins (IL-6, IL-17A), while simultaneously secreting elevated levels of IL-2 and transforming growth factor-β.CD177^+^ neutrophils have been shown to exacerbate endothelial dysfunction through the release of NETs, thereby contributing to the complications associated with cardiovascular disease (CVD) in SLE (10). The mean fluorescence intensity (MFI) of CD177 could serve as a novel biomarker for assessing disease activity in SLE, providing fresh insights for the precise clinical diagnosis and treatment of this condition. Similarly, elevated expression of CD177 on neutrophils in the peripheral blood of patients with rheumatoid arthritis (RA) has been observed (68), alongside increased production of ROS, which can be effectively inhibited by methotrexate (MTX). Although previous studies have suggested that CD177 may serve as a novel indicator for SLE activity or be associated with the pathogenesis of RA, a comprehensive exploration of this biomarker remains lacking. For instance, how does the diagnostic role of CD177 in SLE patients compare to conventional laboratory indicators, such as anti-dsDNA or anti-Smith (anti-Sm) antibodies? Does CD177 help distinguish SLE and RA in clinical? Does CD177 monitor renal injury in SLE? Particularly in the assessment of early renal damage. We look forward to the day when the CD177 monitored by FCM will be used in clinical practice. A summary of CD177 expression across different diseases is shown in **Table 1**.

**Table 1:** A summary of CD177 expression across different tumor.

Disease	Expression level of CD177	Site	The detection method	Prognosis or therapeutic relevance
CRC,gastric	increased	neutrophils	RT-PCR, FCM	a positive correlation with overall survival and disease-free survival,serve as an independent prognostic factor
ovarian cancer	increased	ovarian cancer epithelial tissue	IHC	a negative correlation with poor prognosis
PDAC	increased	in PDAC tissues	GEO database,PCR	High expression of CD177 indicated poor prognosis of PDAC
breast cancer, renal clear cell carcinoma, lung cancer, CRC and melanoma, HCC	increased	Ti Treg cell	RNA-seq, FCM	CD177^+^Ti Treg cells exhibite distinct transcriptional profiles characterized by enhanced immunosuppressive capabilities and were correlated with poor patient prognosis
breast cancer*	reduced	breast epithelial cells	Immunofluorescence, FCM, RT-PCR	lower CD177 levels to predict the poor prognosis
cervical cancer	low expressed	cervical tissues	from GENT2,HPA, GEO database IHC,WB	CD177 as a protective factor
MDS	decreased	neutrophils	FCM	Attributing to specific genetic mutations
COVID-19	upregulated	neutrophils	FCM	CD177 demonstrates high accuracy in identifying patients with severe forms of the disease
COVID-19	also expressed	CD4+ T lymphocyte	FCM	a positive correlation with disease severity
KD and MIS-C	enriched	neutrophils	FCM	Increased CD177 neutrophils are associated with inflammation severity.
AP	increased	neutrophils	FCM	a protective effect of exogenous CD177 for AP
ALI or ARDS	increased	neutrophils	FCM	a protective effect of anti-CD177 for ALI OR ARDS
sepsis, allergic asthma	higher expression	neutrophils	FCM	CD177 is implicated in neutrophil activation and the regulation of inflammatory responses
IBD	increased	neutrophils	PCR	CD177+neutrophils play a protective role through increased bactericidal activity and IL-22 production
			FCM	
		intestinal mucosa	IHC	
			RNA-seq	
SLE,RA	increased	neutrophils	FCM	CD177 MFI-high may serve as a novel biomarker for monitoring disease activity in SLE
noncancer liver diseases	increased	neutrophils	q-PCR,FCM, IHC	It plays a potential therapeutic value

CRC, Colorectal cancer; ESCC, Esophageal squamous cell carcinoma.

HCC, Hepatocellular carcinoma; PDAC, Pancreatic ductal adenocarcinoma.

MDS, Myelodysplastic Syndrome; COVID-19, Coronavirus disease 2019.

KD, Kawasaki disease (KD); MIS-C, Multisystem Inflammatory Syndrome.

AP, Acute Pancreatitis; ALI, Acute lung injury.

IBD, Inflammatory bowel disease; RA, Rheumatoid arthritis.

SLE, Systemic lupus erythematosus; ARDS, Acute respiratory distress syndrome.

FCM, Flow Cytometry; IHC, Immunohistochemistry.

### 4.4 Others

Previous research has established an association between mutations in CD177 and myeloproliferative disease. Overexpression of CD177 has been observed in patients with polycythemia rubra vera and polycythemia. Furthermore, the expression level of CD177 has been closely linked to the severity and prognosis of polycythemia rubra vera (69, 70). Limited studies report the function of CD177+ neutrophils in noncancer liver diseases. CD177^+^ neutrophils exhibited a dominant presence in heatstroke-induced liver injury, which may be involved in regulating NETs (71).Compared with chronic liver disease and healthy controls, CD177 expression was significantly increased in acute-on-chronic liver failure, including *CD177 gene* expression level and CD177 ^+^ neutrophil percentage (72). In addition, CD177^+^ neutrophils were significantly increased in the liver tissue of patients with biliary atresia (36). The use of N-acetylcysteine can reduce the percentage of CD177^+^ neutrophils and NETosis, indicating that it has potential therapeutic value for patients with biliary atresia (73), but the exact function of CD177^+^ neutrophils remains elusive in liver injury.

## 5 The detection methods of CD177

Currently, the primary techniques were used for detecting CD177 include Real-Time Quantitative PCR(qPCR), RNA Sequencing, Flow Cytometry(FCM), Immunohistochemistry(IHC), Immunocytochemistry(ICC), Western Blot(WB). The comparison between methods is shown in **Table 2**. The most reported methods in the literature are FCM and RNA-seq, which are the most recommended methods. qPCR and RNA-seq are quantitative detection methods that are widely used in research experiments. Compared with the former, FCM can not only quantitatively detect CD177, including CD177^+^(%) and CD177^+^MFI, but also distinguish and sort CD177^pos^ and CD177^neg^. Flow cytometry is one of the important detection methods for the study of neutrophils and Treg cells. Personal suggestion with CD45/CD177 double antibody combined panel. According to individual experiments, you can choose multi antibody joint detection, such as CD11b, CD11C, CD3, CD4, etc, and more parameters can be obtained at the same time. However, for the localization of CD177 expression in tumor tissues, IHC or ICC is irreplaceable.

**Table 2:** Comparison of the main CD177 detection methods.

Method	Sensitivity	Specific	Application
qPCR or RNA-Seq	middle	High/extremely high	Quantitative, the operation is more complex, suitable for research level
ICC, IHC	Low	middle	half quantitative,It can locate tissue or cells
WB	high	high	Quantitative,suitable for research, complex operation, time-consuming and many interference factors.
FCM	Single cell expression can be detected	>95%,distinguish and sort CD177^pos^/CD177^neg^cells	Living cell detection,convenient operation, and multi-parameter results can be obtained at the same time.

## 6 The clinical application of CD177

CD177 has shown good prospects in clinical application, especially in the early diagnosis and prognosis evaluation of infections, tumors and autoimmune diseases. For example, CD177 ^+^ neutrophils are increased in patients with sepsis, which may be a biomarker for assessing the severity of infection. The role of CD177 in the tumor microenvironment has also attracted much attention, and it may become as a new indicator to evaluate tumor progression and patient prognosis. The prospect of CD177^+^ MFI as a diagnostic or prognostic marker for systemic lupus erythematosus. To expand its applications, multi-center clinical trials were conducted to verify the clinical diagnostic value of CD177^+^ neutrophils as an inflammatory marker for specific diseases, including assessing the ratios of CD177^+^ neutrophils to total neutrophils, CD177^+^ neutrophils to lymphocytes, and CD177^+^ neutrophils to high-density lipoprotein, which may serve as cut-off points for determining the state of infection. In addition, the targeted application of anti-CD177 in tumors necessitates validation through animal experiments and future clinical trials. However, there are still challenges persist, primarily manifesting in the obstacles and specific standardization needs of translational medicine. Currently, the detection method and standardized protocols for CD177 are not fully established, which may result in variability of results across different laboratories. To address this issue, we recommend using flow cytometry as a universal detection method to establish a standardized experimental protocol, including the development of a comprehensive panel, detailed operational steps, selection of fluorescent antibodies and instruments, evaluation of interfering factors, and performance verification, which is conducive to improving the credibility of laboratory research results. Furthermore, the biological function of CD177 and its potential clinical applications will require support from multi-center, large-scale, prospective clinical research in the future.

## 7 Conclusion

In summary, the expression of CD177 on neutrophils is significantly increased in the context of inflammation, autoimmune diseases, and certain tumors, correlating with the severity of inflammation and tumor prognosis. During the progression of inflammation, the body responds to inflammatory stimuli, leading to a rapid increase and activation of CD177^+^ neutrophils, which accumulate at the sites of inflammation as a defensive mechanism, playing a crucial role in antimicrobial activities and barrier protection. Dysregulation of CD177^+^ neutrophils, resulting in immune overactivation, is a key mechanism in autoimmune diseases, and the mean fluorescence intensity (MFI) of CD177^+^ neutrophils may serve as a novel biomarker for assessing disease activity in SLE. Furthermore, CD177 is also expressed on tumor-infiltrating regulatory T cells (Ti Treg) and within tumor tissues, indicating a dual role in influencing the clinical and prognostic values across various cancers (**Table 1**). Therefore, CD177 may emerge as a new therapeutic target for certain tumors. However, the functions of CD177 remain incompletely understood. Despite limited studies that have primarily identified variations in CD177 expression and its correlation with diseases in the context of tumors, inflammation, and autoimmune disorders, there is a lack of in-depth mechanistic research and large-scale, multicenter prospective studies for validation. Given the critical role of CD177 in various diseases and the current gap in understanding its mechanisms, future comprehensive research on CD177 may provide new insights for disease diagnosis and clinical treatment.
